# Alumni Association, Buffalo Medical College

**Published:** 1881-02

**Authors:** 


### ALUMNI ASSOCIATION, BUFFALO MEDICAL COLLEGE.

THE annual meeting of the Alumni takes place at the College, on Feb. 21st. The Executive Committee have prepared an excellent programme, and it is expected there will be this year an unusually large attendance of the graduates of the college.

The association will meet at 11 A. M. for business, and all our readers will rejoice to learn that Prof. Miner's health has so far improved that it is confidently expected he will be able to deliver the address of welcome to the Alumni on behalf of the Faculty.

The proceedings at the afternoon session will be confined to matters of scientific interest.

In addition to the annual address of the President, a paper will be read by Prof. C. A. Doremus, Ph. D., M. D., in elucidation of some *chemical points regarding albuminuria.* The lecture will be illustrated by sections of the kidney and other microscopic specimens, enlarged and projected, by means of the electric light and gas microscope. The second paper will be presented by Bernard Bartow, M. D., on the *use of leather for surgical appliances.* This lecture will also be illustrated by clinicial cases demonstrative of the process, etc. The well known experience of both these gentlemen, upon the special subjects selected, ensure papers of far more than ordinary value.

The evening session will be at St. James Hall in connection with the commencement exercises of the College. Prof. Moore will deliver the address to the graduating class. We are glad to learn that the candidates for graduates are not only more numerous, but also, as a class, better qualified in every way for the honorable position to which they aspire.

It gives us great pleasure to be able to announce that the address to the alumni will be delivered by the Right Rev. A. Cleveland Coxe, Bishop of Western New York.

The proceedings will be terminated by a banquet at the Tifft House.

